# Electricity in the Diseases of Women

**Published:** 1891-03

**Authors:** 


*Electricity in the Diseases of Women.* By G. BETTON MASSEY, M.D. Second Edition. Philadelphia: F. A. DAVIS. 1890.

This little work is a very good one to instruct a medical man in the use of electricity in certain diseases of women; but for anyone who has already mastered the elements of electricity, the greater part of the first 100 pages may be passed over. In reference to the apparatus recommended, we are of opinion that a much more simple form of constant-current battery can be purchased of any good English maker. We are in the habit of using an acid sulphate of mercury battery for constant current, and find it fulfils all requirements. Again, a much more simple form of rheostat answers all purposes.

In the relief of stenosis, the author of this work relies on the use of the *positive* pole, whereas we have always found that the *negative* pole produces a much larger canal; and, in fact, we frequently have to use the negative pole to overcome the contraction brought about by the positive pole when treating fibroids of the uterus. The general good results obtained in the treatment of myoma uteri by Dr. Massey is in accordance with the opinion of men in this country and in France who have thoroughly tested the treatment. The author states he has had no experience with the Faradic current in inflammatory conditions of the appendages, preferring the Galvanic current instead. We, however, consider the Faradic preferable to the Galvanic in these cases, and use it by means of a bi-polar vaginal electrode. Dr. Massey's use of the intra-uterine galvanic current for pelvic pain is to be regarded in some cases as of very great service, but in the majority of cases the Faradic current is to be preferred. The electrical treatment of extra-uterine pregnancy we condemn absolutely, and consider the treatment advocated by the author as highly dangerous. In amenorrhœa, Dr. Massey has had good results by the use of electricity, the bleeding produced by the negative pole being most marked; but we cannot accept in all cases that menstruation has been established simply because a blood discharge from the uterus has been induced. In carcinoma, the author is of the same opinion as most electricians, that the high currents interrupted are very dangerous, and that all the good results can be obtained by comparatively weak currents used in connection with puncture. We entirely agree with this.

The book as a whole is written in good style and the ideas are clearly expressed, and to those who wish for information on what is a new subject to many we recommend the perusal of the work.

